# 术后随访不容忽视

**DOI:** 10.3779/j.issn.1009-3419.2018.03.17

**Published:** 2018-03-20

**Authors:** 坚 胡

**Affiliations:** 浙江大学医学院附属第一医院 First Affiliated Hospital to Medical College of Zhejiang University

肺癌是全球最常见的恶性肿瘤之一，其发病率和死亡率一直位于所有恶性肿瘤之首，复发和转移是肺癌手术后死亡的主要原因，因此，术后的随访监测显得尤为重要，可以早期发现和治疗复发转移，提高患者的生活质量，改善生存。目前多数临床中心仍是按传统的随访模式进行随访，术后2年内每3个月门诊随访，2年-5年为每半年门诊随访，5年后每年门诊随访。随着影像学技术和医疗水平的提高，薄层低剂量计算机断层扫描（computed tomography, CT）筛查的普及，早期肺癌的发现率越来越高，针对这部分患者手术后的随访目前还缺乏临床研究及相关依据。

本文作者基于其中心416例接受解剖性肺叶切除及系统性淋巴结清扫的Ⅰ期非小细胞肺癌（non-small cell lung caner, NSCLC）行R0切除后的随访结果，发现Ⅰ期NSCLC术后复发转移虽然集中在术后5年之内，但2年内复发转移风险并不高于2年-5年，通过对年龄、性别，是否吸烟、心肺合并症、肿瘤位置、手术方式、病理类型、pT分期及表皮生长因子受体（epithelial growth factor receptor, EGFR）突变情况进行比较，发现pT2a患者复发转移率高于pT1，有术前心肺合并症者5年DFS较无心肺合并症者差。因此建议2年内随访时间间隔无需比2年-5年更频繁，并在随访内容方面建议术后每年1次-2次胸部增强CT检查，5年后改为每年1次，头颅磁共振成像（magnetic resonance imaging, MRI）及骨扫描检查可只在有症状时实施，另外，对高风险者（如T2a）建议给予相对积极和严格的随访，低风险者（如T1）则可以相对保守和宽松。

文章为我们在Ⅰ期NSCLC的随访策略的制定方面提出了不少宝贵建议，并且有相关的数据对结果进行支持，只是正如作者所述，目前该结果还不能直接应用于临床实践，需要更多的前瞻性数据进行验证及对照研究。同时，影响肺癌患者术后复发转移的因素还有很多，需要多因素数据进行细化分层研究，例如第8版肿瘤-淋巴结-转移（tumor-node-metastasis, TNM）分期系统中已将T分期中T1分为T1a、T1b、T1c；在病理分期中分化程度，腺癌中出现某些特定的结构成分（鳞屑样成分、腺泡、乳头状、微乳头状、实体型、黏液性）以及脉管癌栓等，这些因素均为可能引起肺癌患者术后复发转移的高危因素。如果能将影响肺癌患者术后复发转移的诸多因素综合分析并建模，根据已建模型制定肺癌患者术后的个体化随访策略，将是今后早期肺癌随访的重要手段和发展方向，但仍需更多临床数据支持并不断完善。

